# From start to finish: quantitative allergen risk assessment of foods containing peanut advisory labeling

**DOI:** 10.1186/2045-7022-1-S1-S66

**Published:** 2011-08-12

**Authors:** Ben Remington, Joe Baumert, Dave Marx, Steve Taylor

**Affiliations:** 1 University of Nebraska, Lincoln, USA

## 

Foods with allergen advisory/precautionary labeling (i.e. “may contain, processed in the same facility, etc.”) continue to be prevalent and may be increasingly ignored by allergic consumers. We sought to determine the residual levels of peanut in various packaged foods bearing advisory labeling, compare similar data from 2005 and 2009, and determine any potential risk for peanut-allergic consumers. This practical session details exactly how each risk assessment input variable was determined and used within our simulations. To examine peanut-allergic consumers’ risk of an allergic reaction we chose to look at nutrition bar consumption, the product category with the highest levels of peanut in the advisory labeling study. United States consumption data for nutrition bars were extracted from the National Health and Nutrition Examination Survey (NHANES) using a combination of the 2003-04 and 2005-06 surveys. The analytical data from nutrition bar products from the 2005 and 2009 surveys were combined into one group to provide more samples for statistical testing. The probabilistic risk assessment model for food allergens is described and all statistical tests were done in SAS (version 9.2). Probabilistic risk assessment showed the risk of a reaction within the peanut-allergic population from nutrition bar consumption was 4 in 100,000 on a daily basis. However, many foods with advisory labeling did not contain detectable levels of peanut residue and as a result may not warrant the use of advisory labeling. The use of probabilistic modeling would provide the food industry with a quantitative risk assessment method to assist with determining when advisory labeling is appropriate.

